# Supplementary material for: Full publication of preprint articles in prevention research: an analysis of publication proportions and results consistency
Source: Sci Rep. 2023 Oct 9;13:17034. doi: 10.1038/s41598-023-44291-4 (PMC10562443; doi:10.1038/s41598-023-44291-4)
Supplement: Supplementary file 1 — Supplementary Information. [file 41598_2023_44291_MOESM1_ESM.pdf]

Supplementary information

Table S1: Search string for webcrawler

Medarxiv: December 15, 2020 and October 5, 2021
prevention OR preventive OR immunisation* OR immunization* OR vaccination* OR counselling OR counseling OR "health promotion" OR "health education" OR "behaviour change" OR "behavior change" OR "lifestyle intervention" OR screening OR "early detection" OR "early diagnosis" OR chemoprevention OR chemoprophylaxis OR prophylaxis OR prophylactic OR "risk reduction" OR "reduce the risk" OR "reducing the risk"

Table S2: Characteristics of included preprints according to study design

	**Total n/N (%)**	**Covid-19 topic n/N (%)**	**External funding n/N (%)**
Study design			
Diagnostic study	100/329 (30.4%)	73/100 (73.0%)	60/100 (60.0%)
Cross-sectional study	98/329 (29.8%)	72/98 (73.5%)	45/98 (45.9%)
Ecological study	54/329 (16.4%)	46/54 (85.2%)	16/54 (29.6%)
Descriptive study	25/329 (7.6%)	22/25 (88.0%)	9/25 (36.0%)
Cohort study	25/329 (7.6%)	12/25 (48.0%)	15/25 (60.0%)
Before-after study	13/329 (4.0%)	11/13 (84.6%)	3/13 (23.1%)
RCT	7/329 (2.1%)	4/7 (57.1%)	7/7 (100%)
Time series	5/329 (1.5%)	2/5 (40.0%)	3/5 (60.0%)
Case control study	1/329 (0.3%)	0/1 (0%)	1/1 (100%)
Case series	1/329 (0.3%)	0/1 (0%)	1/1 (100%)

RCT, randomized controlled trial;

Table S3: Characteristics of included preprints according to funding source

	**Total n/N (%)**	**Covid-19 topic n/N (%)**
Study design		
Public or non-commercial	98/329 (29.8%)	63/98 (64.3%%)
Public and non-commercial	42/329 (12.2%)	17/42 (40.5%)
Industry	20/329 (6.1%)	11/20 (55.0%)
No external funding	160/329 (48.6%)	144/160 (90.0%)
Not reported	9/329 (2.7%)	7/9 (77.8%)

Table S4: Proportions of published preprints according to study design

	**Published within 12 months n/N (%)**	**Median impact factor (range)**	**Median months until publication (range)**
Study design			
Diagnostic study	32/100 (32.0%)	5.7 (0.2 – 53.4)	6.2 (1.4 – 11.1)
Cross-sectional study	70/98 (71.4%)	3.2 (0.5 – 25.1)	5.1 (-0.1* – 10.8)
Ecological study	17/54 (31.5%)	5.0 (0.8 – 35.5)	5.2 (1.0 – 10.8)

Descriptive study	13/25 (48%)	2.9 (0.2 – 39.9)	5.5 (2.5 – 10.6)
Cohort study	13/25 (52.0%)	3.2 (1.4 – 22.7)	4.8 (0.3 – 11.3)
Before-after study	4/13 (30.8%)	2.6 (2.2 – 6.8)	3.5 (0.2 – 7.0)
RCT	7/7 (100%)	8.3 (2.4 – 74.7)	4.6 (0.9 – 10.8)
Time series	3/5 (60.0%)	2.7 (2.0 – 4.3)	10.4 (0.9 – 10.9)
Case control study	1/1 (100%)	3.1 (NA)	5.2 (NA)
Case series	1/1 (100%)	11.1 (NA)	7.3 (NA)

*Date of publication before first appearance on medRxiv
RCT, randomized controlled trial; NA, not applicable;

Table S5: Proportions of published preprints according to funding source

	**Published within 12 months n/N (%)**	**Median impact factor (range)**	**Median months until publication (range)**
Funding source			
Public or non-commercial	54/98 (55.1%)	3.2 (0.9 – 53.4)	5.3 (0.5 – 11.2)
Public and non-commercial	32/42 (76.2%)	5.3 (0.7 – 59.1)	6.2 (-0.1 – 9.8)
Industry	10/20 (50.0%)	4.3 (2.4 – 74.7)	5.7 (0.9 – 11.0)
No external funding	61/160 (38.1%)	2.7 (0.2 – 13.1)	4.7 (0.2 – 11.0)
Not reported	4/5 (80.0%)	2.2 (1.1 – 2.3)	5.1 (1.6 – 6.3)

Figure S1: Prevention type as predictor for publication of preprints (probability expressed as %, 95% credible intervals)

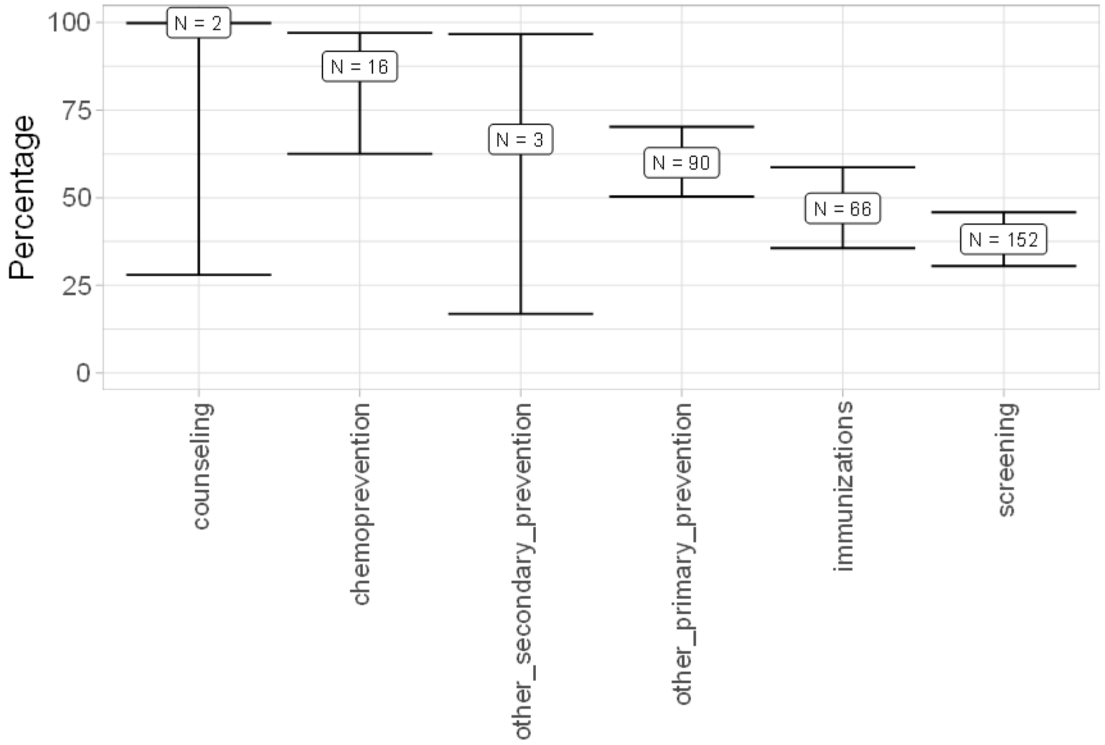

Figure S2: COVID-19 yes/no as predictor for publication of preprints (probability expressed as %, 95% credible intervals)

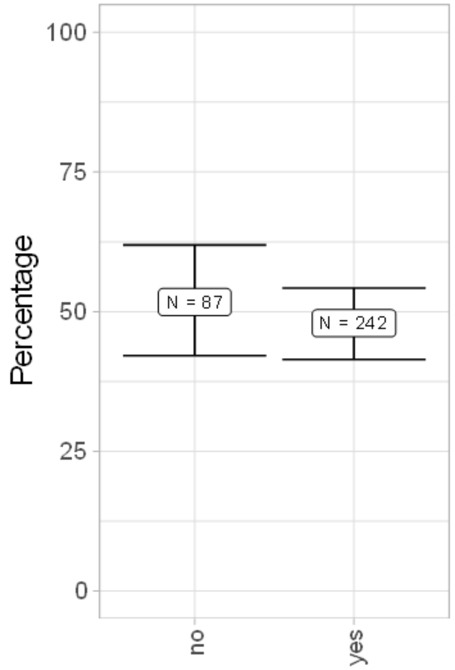

Figure S3: Study design as predictor for publication of preprints (probability expressed as %, 95% credible intervals)

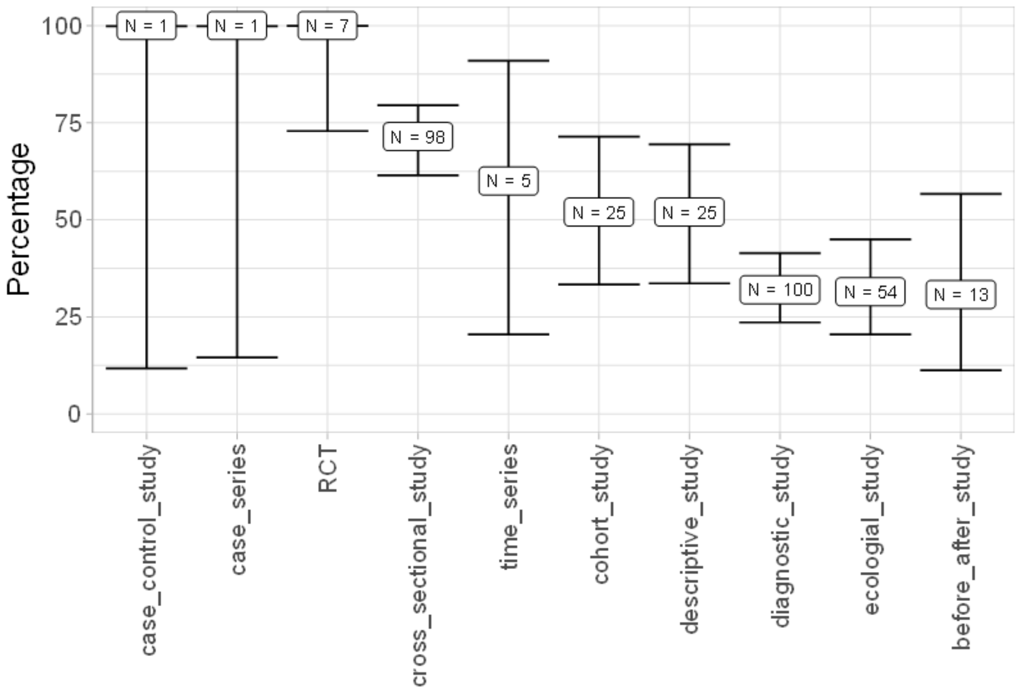

Figure S4: Funding source as predictor for publication of preprints (probability expressed as %, 95% credible intervals)

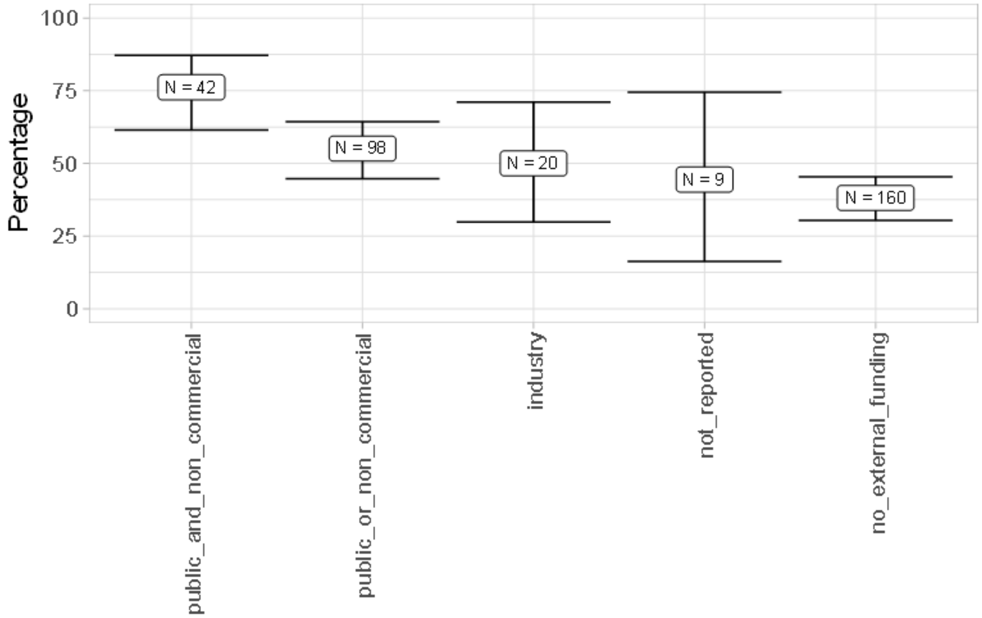

Table S6: Sample characteristics of authors of non-published preprints (n=12)

**Author characteristics**	Percentage	Numbers
**Age in years**		
20-29	8.3%	1
30-39	33.3%	4
40-49	16.7%	2
50-59	25.0%	3
60-69	8.3%	1
70-79	8.3%	1
**Gender**		
Male	91.7%	11
Female	8.3%	1
**Country of work**		
China	8.3%	1
India	25.0%	3
Nigeria	8.3%	1
Pakistan	8.3%	1
Philippines	8.3%	1
UK	8.3%	1
USA	33.3%	4
**Current position**		
Student	8.3%	1
Assistant/Associate Professor	16.7%	2
Professor	16.7%	2
Researcher in academia/industry/ non- academic organizations	33.3%	4
Clinician	8.3%	1
Other:<br>1. Chief Medical Officer of Health of a district<br>2. Limited Liability Company member	16.7%	2
**Years of experience in research**		
1-10	33.3%	4
11-20	41.7%	5
21-30	16.7%	2
>30	8.3%	1

Table S7: Survey results (n=12)

Question item	Percentage	Numbers
**Reasons for not publishing the preprint in a peer-reviewed journal within one year after being uploaded to *medRxiv***		
Lack of time	25.0	3
Rejection by ≥ 1 journal	58.3	7
Other:<br>1. It has received its due attention<br>2. No intention of peer-reviewed publication.	16.7	2
**Number of times the paper was rejected (if it was rejected) (n=7)**		
1-2	28.6	2
3-4	57.1	4
5-6	14.8	1
**Official reason for rejection of the paper in a peer-reviewed journal (multiple answers possible)**		
Too few figures/tables	NA	2
Journal’s scope was not met	NA	2

Lack of novelty	NA	3
Inappropriate study design	NA	1
Writing style	NA	1
Poor language quality	NA	1
Too many incoming manuscripts	NA	2
Other:	NA	4
1. Word count exceeded		1
2. No reason given		2
3. Not sufficient interest		1
**Funding source of non-published preprint**		
Commercial (industrial)	8.3	1
Governmental	25.0	3
Non-governmental	0	0
Mixed	0	0
No external funding	66.7	8
**Reasons for uploading preprint (multiple answers possible)**		
Sharing results with the community	NA	11
Immediate/fast publication	NA	6
Increased visibility of work	NA	6
Other:	NA	1
1. COVID		
**Numbers of preprints having uploaded in the past**		
1	25.0	3
2-5	50.0	6
More than 5	25.0	3
**Number of preprints having published in a journal in the past**		
0	33.3	4
1	33.3	4
2-5	16.7	2
More than 5	16.7	2
**"Preprints have an appropriate level of credibility."**		
Strongly agree	16.7	2
Agree	33.3	4
Undecided	33.3	4
Disagree	16.7	2
Strongly disagree	0.0	0
**Do you cite preprints of other authors who do not get published in peer-reviewed journals in your manuscripts?**		
Often	25.0	3
Sometimes	41.7	5
Seldom	16.7	2
Never	16.7	2

NA, not applicable
